# The Royal Victoria Hospital, Belfast

**Published:** 1907-08-03

**Authors:** Conrad W. Thies

**Affiliations:** Secretary Royal Free Hospital, London


### The Hospital Secretary's Column

[Contributions to this Column are invited, and if accepted will be paid for.]

### THE ROYAL VICTORIA HOSPITAL, BELFAST.

By Conrad W. Thies, Secretary Royal Free Hospital, London.

I was very glad to avail myself of the opportunity recently afforded me during a visit to Belfast to inspect the Royal Victoria Hospital, which was opened by the King and Queen in July, 1903.

This hospital is quite unique and original in design and equipment, and, as was stated by one writer, has made " a revolution in hospital construction." When it was first projected the Committee decided that it should be ventilated and heated upon the " plenum " system, and the architect selected was Mr. William Henman, F.R.I.B.A., who a few years previously had designed and erected the New General Hospital in Birmingham on this principle; it ought, however, to be borne in mind that the general plan of the Birmingham Hospital was designed before it was decided that the buildings should be mechanically ventilated, and this necessarily entailed considerable modifications of the original design and gave rise to many constructive difficulties.

In the case of the Royal Victoria Hospital, however, the " plenum " system was decided upon from the first, and Mr. Henman, who was assisted by Mr. Henry Lea, M.Inst.C.E., as the Consulting Engineer, was given an entirely free hand. The new hospital was, therefore, specially designed with a view to this method of ventilation and heating; it is without any windows that will open or any fireplaces in its wards and working departments, and entirely dependent upon the " plenum " system.

The question of the best method of ventilating hospitals has been and is still the subject of much controversy, and there is probably no subject upon which experts in hospital construction hold such divergent opinions.

As long as we continue to erect our hospitals in the midst of large centres of population, the problem of how the patients can be supplied with the purest air that is available must be considered. This most important aspect of hospital construction was almost entirely ignored at the period when most of our older hospitals were planned, but during the past few years the increasing density of our urban populations and the consequent fouling of the atmosphere of our great cities has forced it upon the attention of architects and engineers, with the result that nearly all the variations in the plans of modern hospitals have been mainly brought about by the desire to ensure efficient ventilation. To the present no agreement has been reached by the experts, the controversy still continues as to the relative merits of what has been termed " mechanical " as opposed to " natural " means for securing this efficiency, the importance of which is now fully recognised by all hospital authorities.

I shall not attempt any detailed description of the Royal Victoria Hospital buildings, etc.; those who are interested in such details will find them fully described in an article which was published, together with the plan, in The Hospital of June 22, 1901. Suffice for my present purpose to say that the seventeen wards are built on the pavilion plan, side by side in a solid block without any intervening spaces, the lighting being by lantern windows on either side of the sloping roofs.

The wards are connected by a corridor 450 feet long by 9 feet broad, from which branch seventeen short corridors giving access to as many wards, each containing fourteen beds; the eight wards on the east side are for medical cases, the eight wards on the west side for surgical cases, and one for gynæcological cases, while two wards are provided on the north side for ophthalmic cases.

The arrangement of the wards is on the unit principle, *i.e.* each physician and surgeon has the control of a male and female ward; to each group of four medical wards a class room is provided, and to each pair of surgical wards is an operating theatre; a ward kitchen serves two wards, and small wards with two beds are provided for special cases. There is ample store room and cupboard accommodation. The lavatories are placed in turrets at the south end of the wards, where there are large glazed doors opening out to balconies.

The hospital is built for 300 beds, but at the present time four wards are still unused owing to lack of funds.

In the administrative blocks excellent accommodation is provided for the resident and nursing staff, and female servants; this block is not built on the " plenum " system, Mr. Henman stating his opinion that while efficient ventilation is secured by the " plenum " system the cost of upkeep is unduly increased by mechanically ventilating a large section of buildings, consisting of numerous small rooms, occupied only for a few hours at a time, which could have been reasonably ventilated by simpler methods, and by opening the windows when the rooms are unoccupied. The temperament of people in health varies so greatly that, when deprived of the right to open or close a window at will, it is next to impossible to persuade them their rooms are efficiently ventilated, and, however good the system may be, some will unreasonably condemn it.

In the wards of a hospital, however, the conditions are entirely different. Occupied throughout both day and night by a number of patients in a low state of health, continuous change of air with an equable temperature and freedom from draughts, secured without noise or dirt, is an ideal state in which health and strength may be regained.

In the article in The Hospital to which I have already referred, the advantages and disadvantages of the " plenum " system are discussed, and the writer makes the following very forcible statement, " that by the result of this system as applied to the Royal Victoria Hospital it must either stand or fall," " for if it does not succeed here it will not be likely to succeed anywhere else."

With these considerations in my mind I visited the hospital in a decidedly critical spirit. I was specially desirous of ascertaining the opinion of persons who had worked in the wards for the period of nearly four years since the hospital was opened.

I was shown over the hospital by Lieut.-Colonel Deane, the Superintendent, who assured me that the practical experience which he had gained of the "plenum" system made him a firm believer in its merits, and he expressed his opinion that it had more than realised the highest expectations of its advocates. I had ample opportunities afforded to me of making enquiries of the sisters and nurses, who had worked in the wards for some time, and they were unanimous in approval of the system. I was informed that the work of keeping the wards clean was reduced to a minimum, owing to the absence of dust and smuts. One sister incidentally mentioned that she had never seen a fly in the wards, and the engineer told me that on occasions, when there was dense fog outside it was possible to see clearly from one end to the other of the long corridor. I found the ward thermometers registered from 58° to 60° F. In all the wards the air was fresh, and even in a surgical ward where there were several foul cases I could not detect any unpleasant odours. I tested the movements of the air by means of a silk handkerchief, and found that although there were no perceptible draughts there was a steady flow of air towards the outlets. I may say that the air is admitted by large openings placed about nine feet from the ground, and is ejected through smaller openings near the floor under each of the beds. It is claimed that the whole of the air is changed every ten minutes in the winter months, and every seven minutes during the summer.

As a result of my visit and inquiries, I feel justified in entirely endorsing the opinion which has been expressed by Professor Byers, one of the hon. medical staff, who, when it was first proposed to build the hospital on the "plenum" system, to place all the wards side by side without intervening spaces, to light them from above, and to have no windows to open, very strongly opposed the proposal, as against all recognised principles of hospital construction; but, after an independent study of the subject and practical experience of its working, has stated that "the hospital has more than fulfilled its expectations, the plenum system of ventilation and heating works admirably: from the point of view of administration and for the comfort of patients I know no hospital equal to it."

I must not conclude these remarks without some reference to the important consideration of economy both in construction and maintenance. As regards construction, it is only necessary to say that the cost of the buildings, including all engineering requirements and a complete steam laundry, was only a trifle over £300 per bed, which I believe is considerably less than that of any other first class hospital in this country. It is much to be regretted that the accounts of the Royal Victoria Hospital are not published in accordance with the Uniform System of Hospital Accounts, as comparison of cost of maintenance with other hospitals is rendered difficult; but as far as I am able to ascertain from the accounts the cost per bed occupied works out at about £52, which is considerably less than that of our best provincial hospitals.

The arrangement of the wards, etc., is most convenient for all administrative purposes, and I was surprised to learn with what a small staff of servants and porters the hospital was worked.

While I still hold to the opinion that in order to obtain the best results from the treatment given to the patients our hospitals ought, as far as is practicable, to be situated on the outskirts of our great cities. I came away from the Royal Victoria Hospital feeling that the "plenum" system had solved the problem how fresh, clean air at the right temperature can be mechanically supplied to the wards of our City Hospitals.

